# The S100A4 Transcriptional Inhibitor Niclosamide Reduces Pro-Inflammatory and Migratory Phenotypes of Microglia: Implications for Amyotrophic Lateral Sclerosis

**DOI:** 10.3390/cells8101261

**Published:** 2019-10-16

**Authors:** Alessia Serrano, Savina Apolloni, Simona Rossi, Serena Lattante, Mario Sabatelli, Mina Peric, Pavle Andjus, Fabrizio Michetti, Maria Teresa Carrì, Mauro Cozzolino, Nadia D’Ambrosi

**Affiliations:** 1 Institute of Anatomy and Cell Biology, Università Cattolica del Sacro Cuore, 00168 Rome, Italy; alessiaserrano@hotmail.it (A.S.); fabrizio.michetti@unicatt.it (F.M.); 2 Department of Biology, University of Rome “Tor Vergata”, 00133 Rome, Italy; Savina.Apolloni@uniroma2.it (S.A.); simona.rossi@ift.cnr.it (S.R.); lab.carri.tv@gmail.com (M.T.C.); 3 Institute of Translational Pharmacology, CNR, 00133 Rome, Italy; mauro.cozzolino@ift.cnr.it; 4 Unità Operativa Complessa di Genetica Medica, Fondazione Policlinico Universitario A. Gemelli IRCCS, 00168 Rome, Italy; Serena.Lattante@unicatt.it; 5 Istituto di Medicina Genomica, Università Cattolica del Sacro Cuore, 00168 Rome, Italy; 6 Unità Operativa Complessa di Neurologia, Fondazione Policlinico Universitario A. Gemelli IRCCS, 00168 Rome, Italy; Mario.Sabatelli@unicatt.it; 7 Centro Clinico NEMO, 00168 Rome, Italy; 8 Istituto di Neurologia, Università Cattolica del Sacro Cuore, 00168 Rome, Italy; 9 Institute of Physiology and Biochemistry “Ivan Djaja”, Faculty of Biology, University of Belgrade, 11000 Belgrade, Serbia; ebolaz@hotmail.com (M.P.); pandjus@bio.bg.ac.rs (P.A.)

**Keywords:** ALS, astrocytes, fibroblasts, microglia, mTOR, neurodegeneration, neuroinflammation, NF-κB, niclosamide, S100A4

## Abstract

S100A4, belonging to a large multifunctional S100 protein family, is a Ca^2+^-binding protein with a significant role in stimulating the motility of cancer and immune cells, as well as in promoting pro-inflammatory properties in different cell types. In the CNS, there is limited information concerning S100A4 presence and function. In this study, we analyzed the expression of S100A4 and the effect of the S100A4 transcriptional inhibitor niclosamide in murine activated primary microglia. We found that S100A4 was strongly up-regulated in reactive microglia and that niclosamide prevented NADPH oxidase 2, mTOR (mammalian target of rapamycin), and NF-κB (nuclear factor-kappa B) increase, cytoskeletal rearrangements, migration, and phagocytosis. Furthermore, we found that S100A4 was significantly up-regulated in astrocytes and microglia in the spinal cord of a transgenic rat SOD1-G93A model of amyotrophic lateral sclerosis. Finally, we demonstrated the increased expression of S100A4 also in fibroblasts derived from amyotrophic lateral sclerosis (ALS) patients carrying *SOD1* pathogenic variants. These results ascribe S100A4 as a marker of microglial reactivity, suggesting the contribution of S100A4-regulated pathways to neuroinflammation, and identify niclosamide as a possible drug in the control and attenuation of reactive phenotypes of microglia, thus opening the way to further investigation for a new application in neurodegenerative conditions.

## 1. Introduction

The small protein S100A4 belongs to the S100 family of EF-hand Ca^2+^-binding proteins, which, upon alterations in intracellular Ca^2+^ concentration, change their conformation and their interaction with target proteins, modulating their activity [1]. S100A4 can control different intracellular pathways leading to a range of effects that are often cell- and tissue-type dependent [2]. It belongs to the very well-known markers characterizing the endothelial-to mesenchymal transition, a composite biological process in which endothelial cells adopt a mesenchymal phenotype, with typical cell morphology and functions, including the acquisition of cellular motility and contractile properties [3]. Indeed, S100A4 is highly expressed in fibroblasts and is a potent regulator of fibrosis in many tissues, such as liver [4], lung [5], heart [6], and tendons [7]. In several types of cancer cells, S100A4 is responsible for their capability to form metastases, promoting their motility and invasiveness [8]. Accordingly, inhibition of S100A4 expression in tumor cells suppresses their metastatic potential, representing, therefore, a strategy to counteract metastatic cancers. Indeed, the S100A4 transcriptional inhibitor niclosamide is currently in phase II clinical trial for metastatic colorectal cancer [9] and completed phase I trial for prostate cancer [10].

Also, highly motile cells as those of the immune system (macrophages, monocytes, T-lymphocytes) express elevated levels of S100A4 [11,12], and, particularly in the case of macrophages, the function of the protein has been linked to cell movement. S100A4^−/−^ mice exhibit no overt abnormalities but show impaired recruitment of macrophages to sites of inflammation in vivo, and accordingly, primary macrophages derived from S100A4-null mice display defects in chemotaxis in vitro [11] and alterations in matrix-degrading capacity [13]. The mechanism of action of S100A4 relies on its interaction with cytoskeletal proteins, such as non-muscle myosin-IIA, tropomyosin, liprin β1, ezrin [14,15,16,17], with consequent changes in cell morphology, adhesion, and migration.

In addition to its intracellular role, S100A4-positive cells can release S100A4 in the extracellular environment where it stimulates pro-inflammatory pathways (including nuclear factor-kappa B (NF-κB)), recruiting immune cells and leading to the secretion of cytokines and proteins remodeling the extracellular matrix, such as matrix metalloproteinases (MMPs) [2].

In the nervous system, S100A4 presence and functions have been studied essentially in models of acute neuronal injury. The protein is expressed by subpopulations of sympathetic and sensory neurons as well as by glial cells in both central and peripheral nervous systems [18]. Its expression is markedly increased in white matter astrocytes that are present in the spinal cord after sciatic nerve or dorsal root resection [19] and influences the formation of a non-permissive glial scar [20]. Genetic deletion of S100A4 exacerbates neuronal loss after traumatic brain injury or excitotoxicity, evidencing, therefore, a protective role for this protein in acute brain stress [21]. In vitro, there are conflicting data about the functions of S100A4 in CNS models. High-molecular weight complexes of the protein stimulate neurite outgrowth in different populations of primary neurons and promote survival [22,23]. On the contrary, in astrocytes-dorsal root ganglion cells co-cultures, S100A4 inhibition stimulates neurite outgrowth, suggesting possible opposite mechanisms exerted respectively by extracellular and intracellular S100A4 [24]. Furthermore, S100A4 seems to have opposite effects also on astrocyte motility, when comparing primary astrocytes to C6 astrocytoma cells [25]. Despite these studies, the role of S100A4 in chronic neurodegenerative disease is still lacking.

In this study, we analyzed the expression of S100A4 in activated primary microglia and the capability of niclosamide to inhibit several pro-inflammatory features. We found that S100A4 was strongly up-regulated in reactive microglia and that niclosamide was able to inhibit NADPH oxidase 2 (NOX2) increase, the activation of NF-κB and mammalian target of rapamycin (mTOR), cytoskeletal rearrangements, migration, and phagocytosis. Furthermore, we investigated the expression and localization of S100A4 in a neurodegenerative disease characterized by a strong neuroinflammatory component, amyotrophic lateral sclerosis (ALS). We demonstrated an increase of S100A4 expression in microglia and astrocytes of the spinal cord from transgenic superoxide dismutase 1 (SOD1)-G93A rats and in fibroblasts derived from ALS patients carrying *SOD1* pathogenic variants, indicating a specific cell type overexpression of S100A4 and suggesting its possible inflammatory function in ALS.

## 2. Materials and Methods

### 2.1. Transgenic Animals

Experiments were performed on wild type (WT) and transgenic Sprague–Dawley male rats, carrying human mutated SOD1-G93A (002148-T, NTac: SD-Tg (SOD1G93A) L26H; Taconic, Hudson, NY, USA). Animals were defined as pre-symptomatic at the age of approximately 7 months with no clinical signs of disease and at the peak of the body weight-time curve. End-stage animals were sacrificed when the atrophy of both hind limbs was detected, accompanied by a significant loss of body mass. All experiments were performed according to the rules for animal care proposed by the Serbian Laboratory Animal Science Association, a member of the Federation of the European Laboratory Animal Science Associations, and approved by the Ethics Committee of the Faculty of Biology, University of Belgrade.

### 2.2. Antibodies

The following primary antibodies were used for immunofluorescence (IF) or western blot (WB): anti-rabbit S100A4 (1:500-IF, 1:1000-WB, Millipore, Burlington, MA, USA), anti-mouse glial fibrillary acidic protein (GFAP) (1:1000-IF, Novus Biologicals, Centennial, CO, USA), anti-rat CD68 (1:500-IF, Abd Serotec, Kidlington, UK), anti-rat CD11b (1:500-IF, Abd Serotec), anti-mouse paxillin (1:500-IF, 1:1000-WB, BD-Biosciences, San Jose, CA, USA), anti-mouse gp91^phox^ (1:1000-WB, BD-Biosciences), anti-rabbit mTOR and phospho-mTOR (1:1000-WB, Cell Signaling, Danvers, MA, USA), anti-rabbit NF-κB and phospho-NF-κB (1:1000-WB, Cell Signaling), anti-GAPDH (1:5000-WB, Millipore). Secondary fluorescent antibodies for IF were: Cy3 Donkey anti-rabbit (1:200), Alexa-Fluor 488 Donkey anti-rabbit (1:200), Cy3 Donkey anti-mouse (1:200), and Cy5 Donkey anti-rat (1:200) from Jackson ImmunoResearch Laboratories (West Grove, PA, USA). Phalloidin (1:200, Sigma Aldrich, Milan, Italy) was used to stain cells’ actin filaments. DAPI (1:1000, Thermo Fisher Scientific, Waltham, MA, USA) was used to stain nuclei. Anti-rabbit and anti-mouse IgG peroxidase-conjugated secondary antibodies (1:2500) were from Bio-Rad Laboratories (Hercules, CA, USA).

### 2.3. Primary Microglia Cell Cultures and Pharmacological Treatments

Primary microglia cultures from the brain cortex were prepared, as previously described [26]. Primary microglia were stimulated with 50 ng/mL tumor necrosis factor-alpha (TNFα, PeproTech, London, UK) or 1 μg/mL LPS (lipopolysaccharide) or 100 μM ATP (Sigma Aldrich) for the indicated time. The pretreatment with niclosamide (Sigma Aldrich) was performed 24 h before inflammatory stimuli.

### 2.4. Protein Extraction, SDS-PAGE, and Western Blotting

Protein lysates collected in RIPA buffer (PBS, 1% Nonidet P-40, 0.5% sodium deoxycholate, 0.1% SDS) were centrifuged for 20 min at 14,000× *g* at 4 °C. Supernatants were assayed for protein quantification with the Bradford detection kit (Bio-Rad Laboratories). Proteins were separated by SDS-PAGE and transferred onto nitrocellulose membranes (GE Healthcare, Chicago, IL, USA). Membranes were blocked in 5% non-fat dry milk and then incubated overnight at 4 °C with the indicated primary antibodies. After rinsing with Tris-buffered saline solution with 0.1% Tween-20 (TBS-T), membranes were incubated for 1 h with the appropriate peroxidase-conjugated secondary antibody, then washed and developed using the ECL chemiluminescence detection system (Roche) or Advance Western blot detection kit (Amersham Biosciences, Buckinghamshire, UK). Densitometric analyses were performed using the ImageJ software program (National Institutes of Health, Bethesda, MD, USA).

### 2.5. Migration Assay

For the migration assay, microglia were seeded in removable culture inserts (Ibidi, Gräfelfing, Germany) and treated with 100 nM niclosamide. After 24 h, the inserts were removed, and the cells were stimulated with TNFα or ATP for 48 h. The bright-field images of the migration assay were photographed at 20× magnification at 0, 24, and 48 h from the inflammatory stimulation. Cell motility was determined by counting the number of cells that migrated inside the gap. Each experiment was carried out in triplicate.

### 2.6. Immunofluorescence Microscopy

Primary cells were fixed for 15 min in 4% paraformaldehyde, permeabilized for 5 min in PBS containing 0.1% Triton X-100. The cells were incubated for 2.5 h at 37 °C with the primary antibody and then stained for 1 h with the appropriate secondary antibody. Tissues samples from WT (n = 3), pre-symptomatic (n = 3), and end-stage SOD1-G93A (n = 3) perfused rats were cryoprotected in increasing concentrations of sucrose (10%, 20%, 30%), frozen and cut in 30 μm sections (CM 1850, Leica, Germany). Immunofluorescence analysis was performed in free-floating with sections in 10% normal donkey serum and 0.3% Triton X-100 for 1 h at room temperature and then incubated with the appropriate antibodies in 2% normal donkey serum and 0.3% Triton X-100 for 48 h at 4 °C. Slides were then incubated with appropriate fluorescent-conjugated secondary antibodies in 2% normal donkey serum and 0.3% Triton X-100 for 3 h at room temperature. In both cases, after PBS washes, slides were incubated with 1 μg/mL 6-diamidino-2-phenylindole (DAPI) and cover-slipped with Fluoromount mounting medium (Sigma Aldrich). Immunofluorescence was analyzed by means of a confocal laser scanning microscope. Samples were analyzed with a Leica TCS SP5 confocal microscope and processed using LAS AF and Adobe Photoshop software (Adobe, San Jose, CA, USA).

### 2.7. Phagocytosis Assay

Microglia cells were cultured into 24-well plates at a density of 30,000 per well. Fluorescent red latex beads (2 μm diameter, L3030, Sigma Aldrich) pre-opsonised in 50% FBS and added to the cells at a concentration of 1 × 10^6^/mL and incubated at 37 °C for 2 h. The cells were then washed with PBS to remove free beads and fixed in 4% paraformaldehyde. After phalloidin and DAPI staining, the number of beads ingested by the cells was determined using a Leica TCS SP5 confocal microscope. For each well, DAPI and phalloidin images were collected (n = 15–20), and analysis was done by ImageJ software.

### 2.8. Real-Time qPCR

For quantitative real-time PCR, RNAs were isolated using TRIzol (Thermo Fisher Scientific) for tissue extraction. RNAs were quantified and reverse-transcribed with random primers by the GoScript Reverse Transcription System (Promega, Madison, WI, USA). qRT-PCR was performed with GoTaq qPCR Green Master Mix (Promega) according to the manufacturer’s instruction. The primers used were: FW GAPDH 5′CTGAGGACCAGGTTGTCTCC3′; RV GAPDH 5′GGAAGAATGGGAGTTGCTGT3′; FW S100A4 5′GCCTAGCTTCCTGGGGAGAA3′; RV S100A4 5′ CATCAGCTTCTGGAATGCAGC3′

### 2.9. Primary Fibroblast Cultures

This study was approved by the local ethics committee of Università Cattolica del Sacro Cuore protocol P/740/CE/2012. Written informed consent was provided by all the subjects. The diagnosis of ALS was made according to the revised El Escorial/Airlie House Criteria [27]. Patients underwent a 4-mm punch skin biopsy at the distal leg. Skin samples were cut into pieces of approximately 1 mm in diameter on Petri dishes and maintained in BIO-AMF-2 (Biological Industries, Cromwell, CT, USA) culture medium. Explants were transferred to the cell culture flask with a needle, with 3 mL of BIO-AMF-2. The flask was sealed and placed in a 37 °C oven to grow the fibroblasts. The culture medium was changed every 3 days. When cells from the explants presented adequate growth, fibroblasts were detached from the bottom of the flask with a trypsin-EDTA solution (Lonza, Basel, Switzerland) and transferred to 2 new sterile flasks to which 3 mL of BIO-AMF-2 medium was added. This procedure continued until enough confluent flasks were obtained for collecting. Cells were maintained in DMEM containing 1% antibiotics and 20% FBS. Pathogenic variants in the *SOD1* gene were previously identified by standard Sanger sequencing on genomic DNA extracted from patients’ blood samples [28]. Clinical and demographic features of mutated SOD1 patients are summarized in Table 1.

### 2.10. Statistical Analysis

Data are presented as mean ± standard error of the mean (S.E.M). Statistical differences between the two groups were verified by Student’s *t*-test. One-way analysis of variance (ANOVA) followed by Post Hoc Tukey’s test was used for multiple comparisons. The software package MedCalc (Medcalc Software, Mariakerke, Belgium) was used for all statistical analyses with differences considered significant for *p* < 0.05.

## 3. Results

### 3.1. S100A4 is Up-Regulated in Microglia by Inflammatory Stimuli, and Niclosamide Inhibits Different Activation-Related Parameters

**Figure 1:**
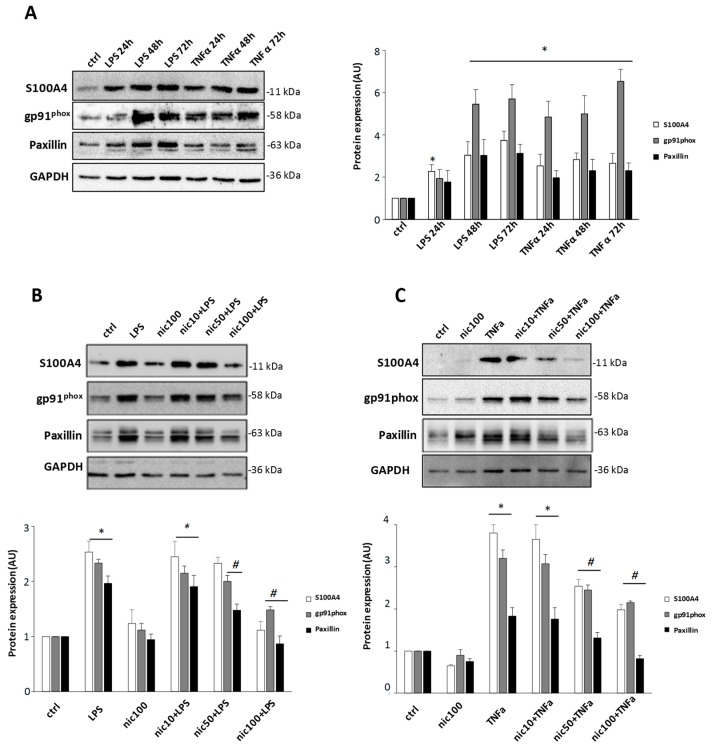
Niclosamide prevents the increase of S100A4, gp91^phox^, and paxillin in LPS- and TNFα stimulated primary microglia. (**A**) Primary microglia were treated with 1 μg/mL LPS or 50 ng/mL TNFα for 24, 48, or 72 h or non-treated (ctrl). Protein lysates were analyzed by western blot using anti-S100A4, anti-gp91^phox^, anti-paxillin, and anti-GAPDH antibodies. Primary microglia were pre-treated with DMSO (ctrl) or 10 nM, 50 nM, 100 nM niclosamide (nic). After 24 h, cells were stimulated with 1 μg/mL LPS (**B**) or 50 ng/mL TNFα (**C**) for 48 h. Protein lysates were analyzed by western blot using anti-S100A4, anti-gp91^phox^, anti-paxillin, and anti-GAPDH antibodies. Quantification of protein bands normalized to GAPDH and relative to control is expressed in an arbitrary unit (AU). Data are reported as mean ± S.E.M. (n = 3 independent experiments). Statistical significance was calculated by ANOVA followed by Post Hoc Tukey’s test. * *p* < 0.05 relative to control (**A**–**C**), # *p* < 0.05 relative to LPS (**B**) or TNFα (**C**).

To investigate whether S100A4 is involved in the acquisition of a proinflammatory phenotype in microglia, we stimulated primary mouse cultures with two different stimuli: lipopolysaccharide (LPS) and TNFα. Cells were treated with TNFα (50 ng/mL) or LPS (1 μg/mL) for 24, 48, and 72 h. We observed an increase of S100A4 starting from 24 h with both stimuli, and elevated levels of the protein were maintained during the time window analyzed. In parallel with this increase, gp91^phox^, a subunit of NOX2, the main ROS source in microglia, and the cytoskeletal adaptor protein paxillin were overexpressed following both treatments (Figure 1A). Based on the strong increase of S100A4 found in active microglia, we next investigated the effect of niclosamide pretreatment on the same parameters. The compound, without interfering with cell viability at the maximum concentration used (100 nM, data not shown), inhibited S100A4 increase starting at the dose of 50 nM after treatment with TNFα and provided a significant inhibition at 100 nM upon LPS stimulation (Figure 1B,C). Remarkably, the inhibitory effect of niclosamide was exerted also on gp91^phox^ and paxillin (Figure 1B,C), whose expression significantly decreased after 100 nM niclosamide pretreatment. These data suggest that S100A4 could be recruited in microglia by different inflammatory stimuli and that the S100A4 inhibitor niclosamide was able to significantly reduce S100A4 expression and several activation-related parameters, such as NOX2 and paxillin.

### 3.2. Niclosamide Prevents Cytoskeletal Rearrangements of Active Microglia

**Figure 2:**
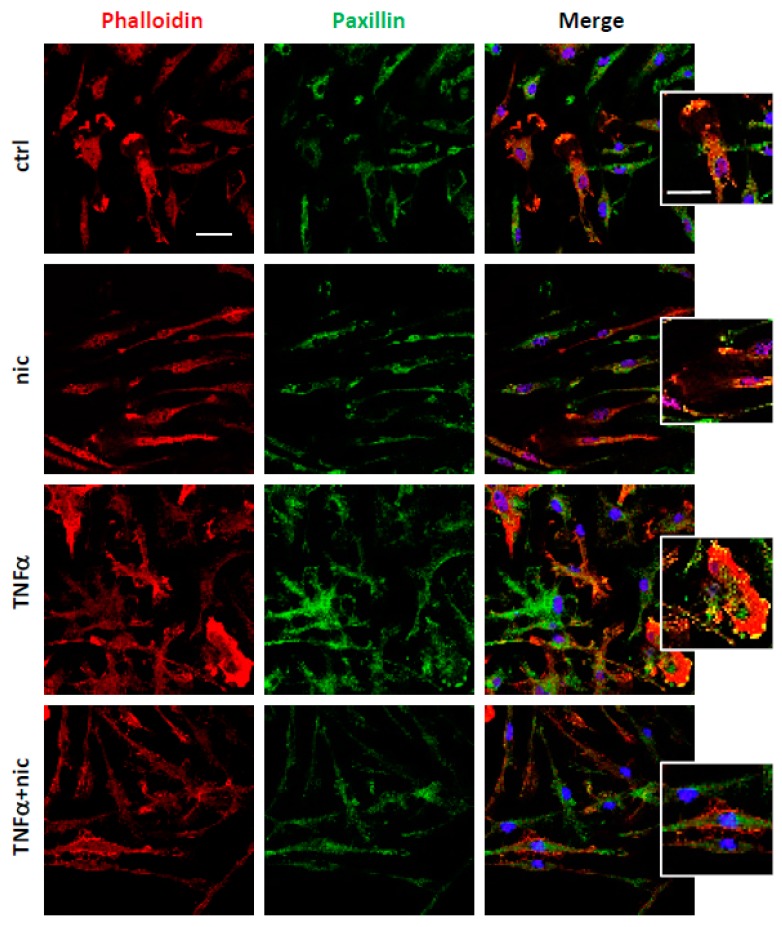
Niclosamide promotes the resting phenotype in tumor necrosis factor-alpha (TNFα)-stimulated microglia. Double immunofluorescence with phalloidin (red) and paxillin (green) on cells treated with DMSO (ctrl) or 100 nM niclosamide (nic) for 72 h. The stimulation with 50 ng/mL TNFα was induced after 24 h from DMSO or niclosamide pre-treatment and left for the following 48 h. In merge images, the nuclei were stained with DAPI. Scale bar: 20 μm. Higher magnification images show lamellipodia. Scale bar: 20 μm.

Since the expression of S100A4 is associated with actin cytoskeleton reorganization and focal adhesion rearrangements in different cell types, among which are fibroblasts and macrophages [8], we then examined whether treatment with niclosamide (at 100 nM) could alter microglia morphology, that was monitored by staining with phalloidin, a probe for actin cytoskeletal filaments, and by analyzing the distribution of paxillin, a marker of focal adhesion complexes. Our results revealed that the changes in cell morphology and cytoskeletal organization, which were observed with TNFα, were suppressed by the treatment with niclosamide (Figure 2), which re-established a phenotype closer to resting cells. In addition, the lamellipodia-like structures containing paxillin found in TNFα-treated cells were also inhibited by niclosamide (Figure 2), suggesting that the compound could interfere with cytoskeletal dynamics.

### 3.3. Niclosamide Modulates Microglia Migration and Phagocytosis Capacity

**Figure 3:**
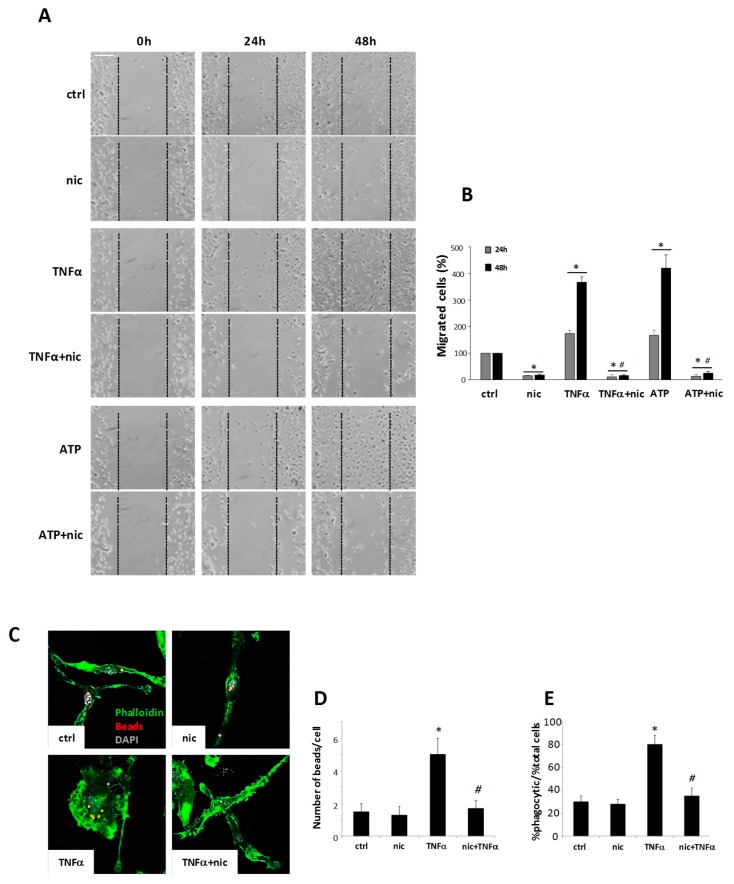
Niclosamide inhibits the migration and phagocytosis of TNFα-stimulated microglia. (**A**) Bright fields images of migration assay at 0, 24, or 48 h from the removal of the mini-chambers insert. Primary microglia inside the mini-chambers were pre-treated with DMSO (ctrl) or 100 nM niclosamide (nic) and after 24 h left free to migrate. After the removal of the mini-chambers inserts, microglia were stimulated with 50 ng/mL TNFα or 100 μM ATP. Scale bar: 150 μm. (**B**) Quantification of migrated cells (%) into the gap after 24 or 48 h from the removal of inserts. (**C**) Fluorescence images of phalloidin (green) and latex beads (red) on cells treated with DMSO (ctrl) or 100 nM niclosamide (nic) or 50 ng/mL TNFα for 48 h. DAPI was used for nuclei staining. Scale bar: 20 μm. The number of beads/cell (**D**) and the % of phagocytic cells/% of total cells (**E**) were calculated. Data represent mean ± S.E.M. (n = 3 independent experiments). Statistical significance was calculated by ANOVA followed by Post Hoc Tukey’s test. * *p* < 0.05 relative to ctrl, # *p* < 0.05 relative to TNFα (**B**-**D**-**E**) or ATP (**B**).

The formation of focal adhesion complexes containing paxillin could be related to the migratory cell capability [29], and thus we analyzed whether niclosamide could also interfere with cell mobility. We demonstrated that niclosamide pretreatment (used at 100 nM) was able to consistently reduce both the spontaneous microglia migration, as well as motility stimulated by TNFα and ATP (Figure 3A,B), a strong inducer of microglia chemotaxis [30]. Furthermore, as focal adhesion proteins and cytoskeletal rearrangements are also implicated in phagocytosis [31], we examined whether niclosamide could modulate the engulfment of fluorescent latex beads by activated microglia. We demonstrated that TNFα led to a significant increase in the number of phagocytic cells, as well as in phagocytic activity per cell, and both of these parameters were strongly inhibited by niclosamide (Figure 3C–E). Thus, these results indicate that niclosamide remarkably decreased both migration and phagocytic capacity of reactive microglia.

### 3.4. Niclosamide Inhibits TNFα-Induced mTOR and NF-kB Expression

**Figure 4:**
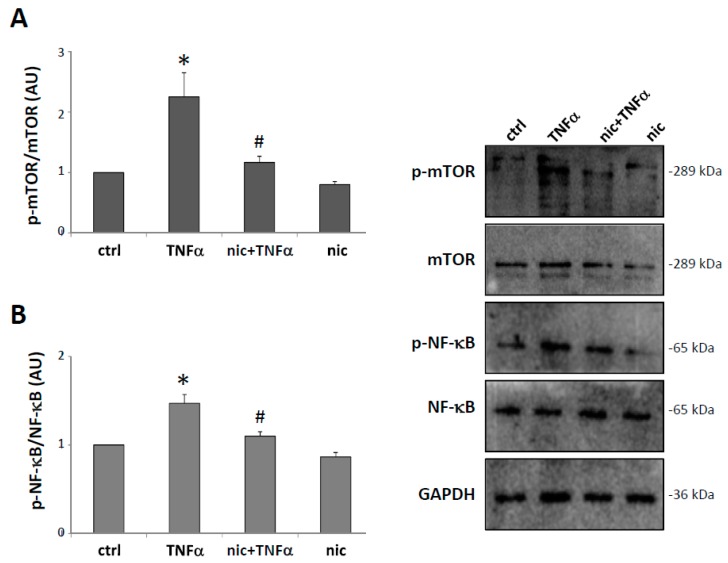
Niclosamide prevents mTOR (mammalian target of rapamycin) and NF-κB (nuclear factor-kappa B) activation in TNFα-stimulated microglia. Primary microglia were pre-treated with DMSO (ctrl) or 100 nM niclosamide (nic). After 24 h, the cells were stimulated with 50 ng/mL TNFα for 48 h. Protein lysates were analyzed by western blot using anti-phospho-mTOR and anti-mTOR (**A**) or anti-phospho-NF-κB and anti-NF-κB (**B**). Anti-GAPDH was used for protein normalization. Quantification of protein bands relative to the control is expressed in an arbitrary unit (AU). Data are reported as mean ± S.E.M. (n = 3 independent experiments). Statistical significance was calculated by ANOVA followed by Post Hoc Tukey’s test. * *p* < 0.05 relative to control, # *p* < 0.05 relative to TNFα-treated cells.

mTOR and NF-κB signaling are common pathways characterizing reactive microglia [32] and are also well-known targets of niclosamide [33,34]. We, therefore, analyzed in microglia the activation of mTOR and NF-κB after TNFα stimulation, in the presence or absence of niclosamide. As shown in Figure 4A,B, TNFα induced an up-regulation of the phosphorylated form of mTOR and NF-κB, and this activation was prevented by pre-treatment with niclosamide, indicating that the compound can inhibit these inflammatory responses in activated microglia.

### 3.5. S100A4 Is Up-Regulated in SOD1-G93A Transgenic Model of ALS

**Figure 5:**
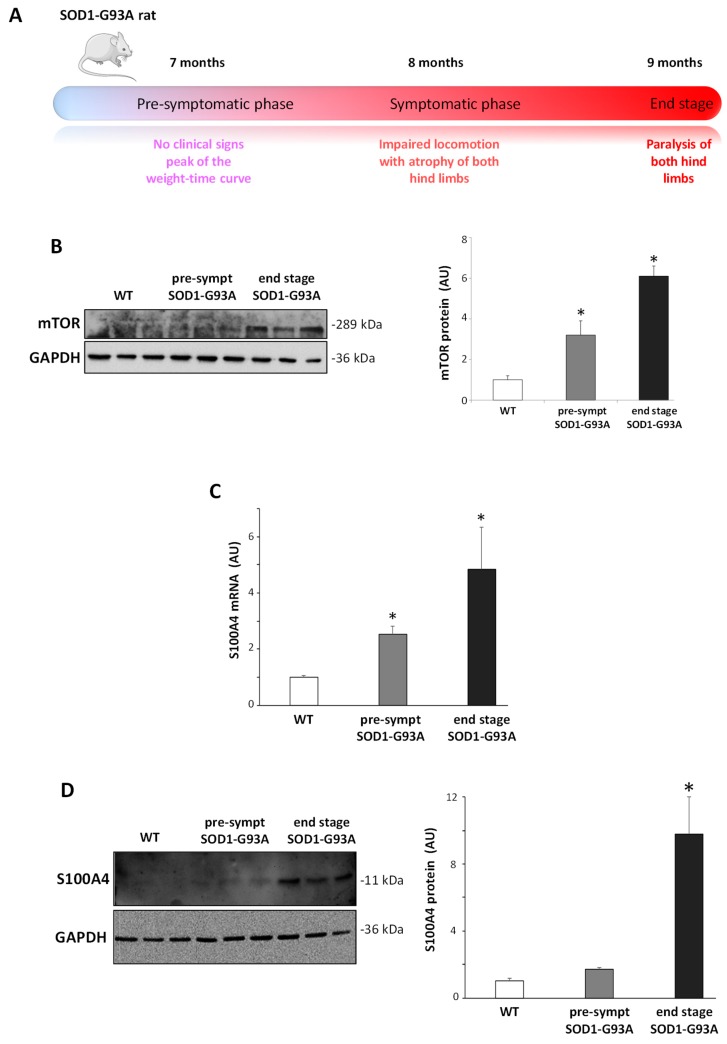
Increase in the S100A4 mRNA and protein in the lumbar spinal cord from pre-symptomatic and end-stage SOD1-G93A rats. (**A**) Schematic illustration of the disease development and progression in the SOD1-G93A transgenic rats. (**B**) Protein lysates of the lumbar spinal cord from wild-type (WT), pre-symptomatic, and end-stage SOD1-G93A rats were analyzed by western blot with the anti-mTOR antibody. (**C**) mRNA from the lumbar spinal cord from WT, pre-symptomatic, and end-stage SOD1-G93A rats was analyzed by RT-qPCR, and quantification expressed in arbitrary units (AU), relative to WT animals. (**D**) Protein lysates of the lumbar spinal cord from WT, pre-symptomatic, and end-stage SOD1-G93A rats were analyzed by western blot with the anti-S100A4 antibody. Anti-GAPDH antibody was used to normalize the samples. The graph shows signal quantification expressed in arbitrary units (AU), relative to WT animals. Data are reported as mean ± S.E.M. (n = 3 animals per group). Statistical significance was calculated by ANOVA followed by Post Hoc Tukey’s test. * *p* < 0.05 relative to WT group.

In order to investigate S100A4 expression in a chronic in vivo neuroinflammatory condition, we adopted transgenic SOD1-G93A rats (Figure 5A), a model recapitulating the pathological features of ALS [35,36]. Interestingly, at both pre-symptomatic and end-stage phases of the disease, SOD1-G93A animals showed increased levels of mTOR protein in the lumbar spinal cord with respect to WT rats (Figure 5B). We next evaluated the levels of S100A4, and, while WT rats did not show age-related differences in the expression of S100A4 (not shown), in SOD1-G93A rats, both S100A4 mRNA (Figure 5C) and S100A4 protein levels (Figure 5D) appeared to be up-regulated at pre-symptomatic phase and further increased at end-stage of the disease.

**Figure 6:**
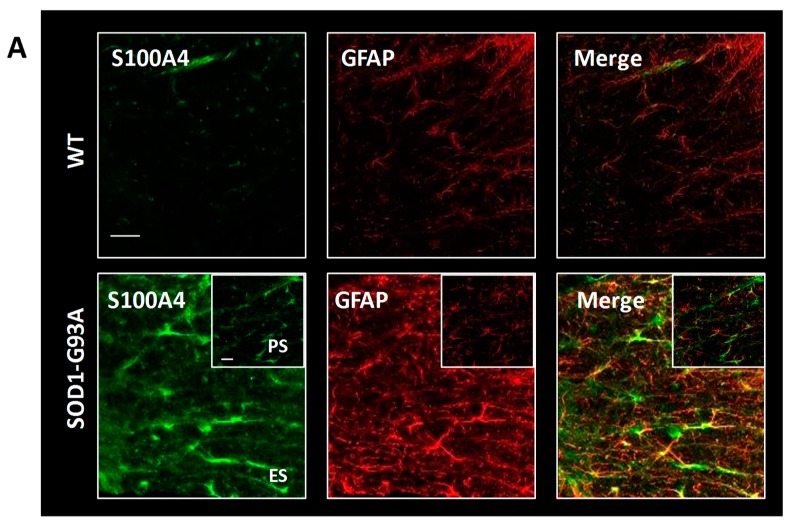

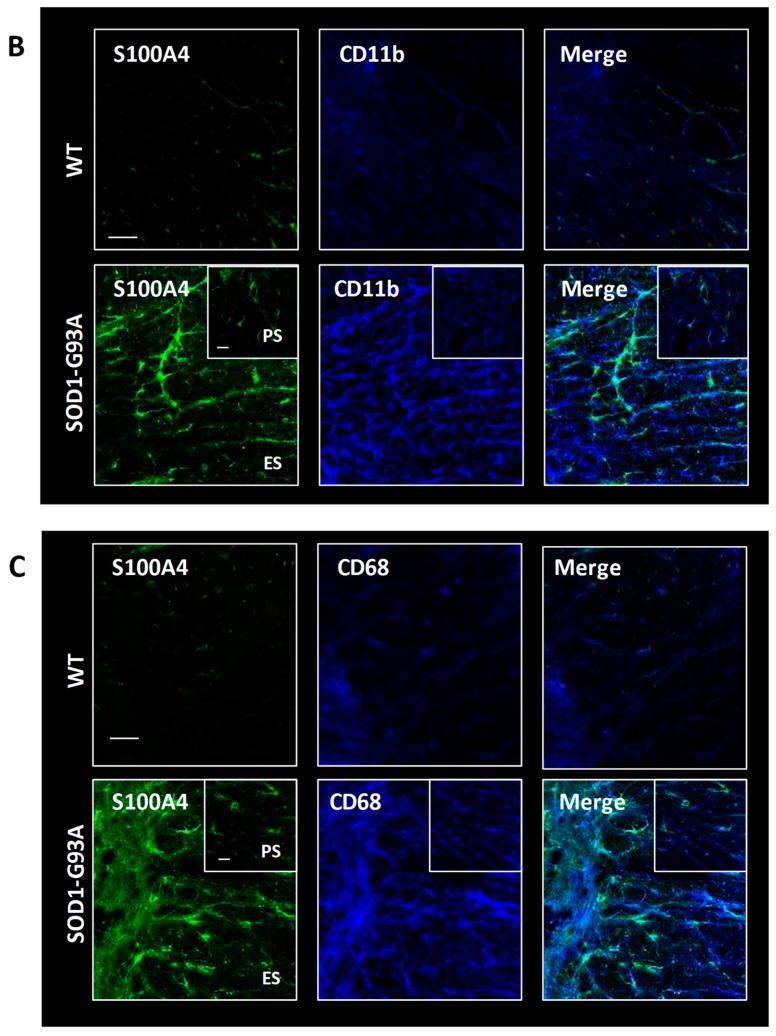
S100A4 increases in astrocytes and microglia/macrophages in the lumbar spinal cord from SOD1-G93A rats. Representative images of double immunofluorescence confocal analysis with anti-S100A4 (in green) and anti-GFAP (in red, (**A**)), anti-CD11b (in red, (**B**)), and anti-CD68 (in red, (**C**)) in the grey and white matter of the lumbar spinal cord from wild-type (WT) and end-stage (ES) SOD1-G93A rats. Insets show images from pre-symptomatic (PS) SOD1-G93A rats. n = 3 animals per group. Scale bars: 50 μm.

We then evaluated by confocal immunofluorescence analysis the cellular localization of S100A4 in the lumbar spinal cord sections of SOD1-G93A rats. As shown, S100A4 immunostaining was almost undetectable in the lumbar spinal cord of WT rats, while in end-stage SOD1-G93A rats, it strongly increased in GFAP-positive astrocytes in both grey and white matter (Figure 6A). Remarkably, at this phase, S100A4 also co-localized with CD11b- or CD68-positive cells (Figure 6B,C), thus highlighting its presence on surveilling and activated cells (i.e., microglia, macrophages) in ventral horns of the lumbar spinal cord of diseased rats. Interestingly, S100A4 staining in GFAP and Cd11b/CD68-positive cells was evident already at the pre-symptomatic phase in SOD1-G93A rats (Figure 6A–C, insets).

### 3.6. S100A4 is Up-Regulated in Fibroblasts from ALS Patients Carrying SOD1 Pathogenic Variants

**Figure 7:**
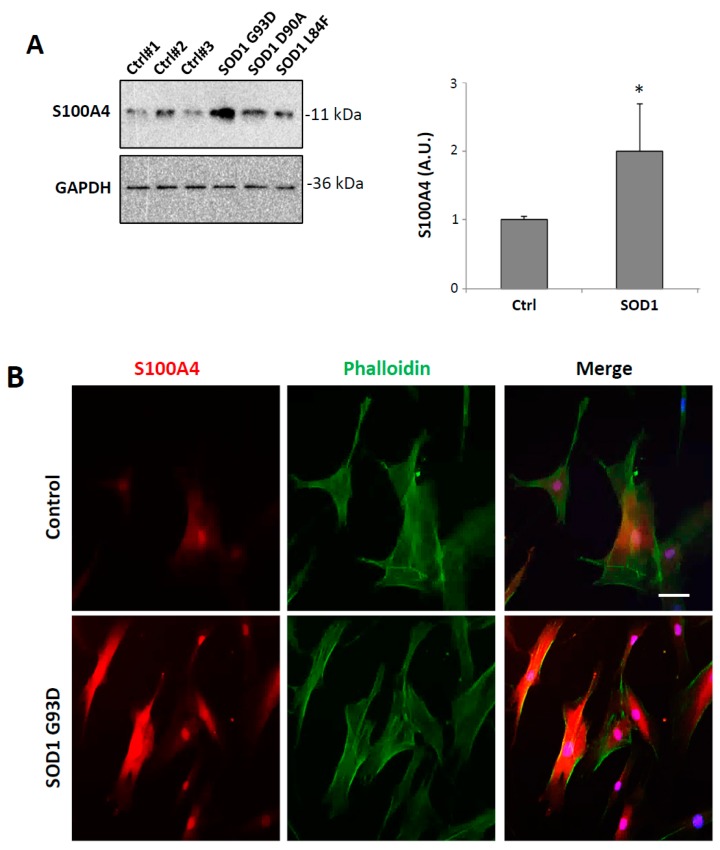

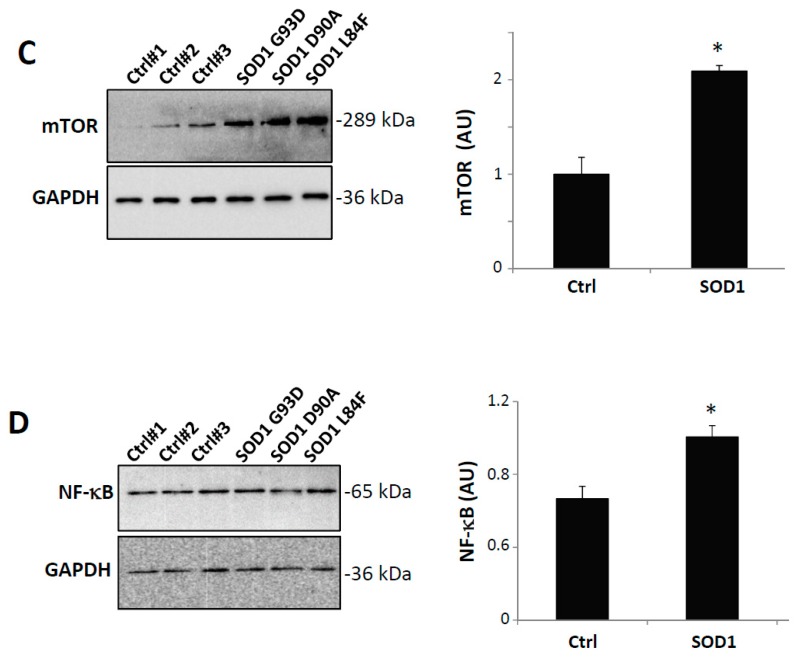
S100A4 is overexpressed in ALS (amyotrophic lateral sclerosis) human fibroblasts harboring SOD1 pathogenic variants. (**A**) Immunoblot analysis of total cell extracts showing increased expression of S100A4 in all patients with SOD1 mutations compared with controls. Anti-GAPDH antibody was used to normalize the samples. (**B**) Representative images of confocal immunofluorescence analysis with phalloidin (green) and anti-S100A4 (red) in a patient with SOD1 G93D mutation. DAPI was used for nuclei staining. Scale bar: 25 μm. Protein lysates of controls and mutant SOD1 fibroblasts were analyzed by western blot using anti-mTOR (**C**) or anti-NF-κB (**D**). Anti-GAPDH antibody was used to normalize the samples. The graphs show signal quantification expressed in arbitrary units (AU), relative to controls. Data are reported as mean ± S.E.M. (n = 3 individuals per group). Statistical significance was calculated by student’s *t*-test. * *p* < 0.05.

Finally, since S100A4 is a protein typically expressed in fibroblasts where it is a potent trigger of pro-inflammatory pathways mediating fibrotic reactions, we sought to analyze its expression in this cell type. To this aim, we compared the expression of S100A4 protein in primary fibroblasts obtained from three ALS patients harboring three different variants in the *SOD1* gene (p.G93D, p.D90A, p.L84F, Table 1) and three healthy donors. As shown in Figure 7A, S100A4 expression was increased in fibroblasts derived from patients with *SOD1* variants (about 2-fold, with respect to control), with a clear overexpression both in the nuclear and cytoplasmic compartments (Figure 7B). The increased levels of S100A4 in mutant SOD1 fibroblasts were concomitant with overexpression of both mTOR (Figure 7C) and NF-κB (Figure 7D) levels, suggesting an alteration of these pathways in peripheral ALS cells, such as fibroblasts from patients with *SOD1* pathogenic variants.

## 4. Discussion

The functions of S100A4 are very well characterized in specific stromal-interstitial cell types, such as fibroblasts, immune cells, and tumor cells [8,37], where it has recently emerged as a potent factor implicated in inflammatory responses, angiogenesis, cell differentiation, apoptosis, motility, and invasion [2]. With this work, we demonstrated that also microglia, representing the immune cells of the CNS, increase the expression of S100A4 upon pro-inflammatory stimuli and that in ALS, a neurodegenerative disease characterized by a strong inflammatory response, both microglia and astrocytes up-regulate S100A4 levels. Our data show that two different pro-inflammatory molecules, with a distinct mode of actions, TNFα and LPS, capable of inducing typical M1 markers, such as NOX2, as well as cytoskeletal rearrangements towards an activated state, both converge on a dramatic and long-lasting up-regulation of S100A4 expression in primary microglia. In parallel with what occurs in macrophages [38,39,40], we could speculate that S100A4 in microglia could be involved both in triggering pro-inflammatory processes as cytokine release, as well as in promoting cellular movement towards a site of injury. In this vein, we have demonstrated that niclosamide, an inhibitor of S100A4 transcription [41,42], can revert the induction of pro-inflammatory parameters as NOX2, NF-κB, mTOR, morphological microglia rearrangements, as well as migration and the phagocytotic activity of microglia. Niclosamide is a drug belonging to the salicylamide family of compounds, and it is widely used in clinical as an antihelmintic. However, in the last years, many high-throughput screenings, aimed at repositioning safe existing drugs, identified niclosamide as an effective compound in the treatment of pathological conditions as cancer, arthritis, viral infections, and fibrotic states [43]. The wide applications of niclosamide may rely on its ability to modulate selected signaling pathways, such as Wnt/β-catenin, mTOR, and JAK/STAT3, which are strongly implicated in these diseases [33,44,45]. Moreover, the wide range of effects of niclosamide may be due to cell type-dependent responses and to the concentration of the compound [43]. We have demonstrated here that in microglia, low doses of niclosamide (in the nanomolar range) are sufficient to inhibit not only S100A4 expression but also reactive microglia responses. Further studies will be necessary to establish whether the effects of niclosamide in microglia are dependent on the specific inhibition of S100A4, and how this, in turn, affects the signaling processes regulated by this protein. Indeed, S100A4 is known to influence not only pathways dealing with cytoskeletal rearrangements, but also NF-κB signaling [38], and to modulate autophagy through interaction with mTOR [46]. Moreover, S100A4 is known to exert its effects by two different mechanisms: an intracellular pathway and an extracellular one [47]. In fact, like all the other members of the S100 family, it can be actively secreted by cells, and in the secreted form, it can act as an alarmin, or bind in an autocrine/paracrine way to different receptors (i.e., receptor for advanced glycation endproducts, toll-like receptor 4), inducing downstream intracellular signaling [7,11,48]. However, regardless of S100A4 mode of action, we have demonstrated here that niclosamide could be a potent inhibitor of microglia reactivity and, therefore, might represent a valuable strategy to attenuate uncontrolled processes of activation as those occurring during neurodegenerative pathologies where excessive microglial activation, migration, and phagocytosis overwhelm their protective function and actively contribute to neuronal damage [49]. Hence, we analyzed if S100A4 was dysregulated also in an in vivo model of ALS, a neurodegenerative disease where the neuroinflammatory aspect is a well-recognized etiopathogenic mechanism. ALS is a multifactorial disease where motor neuron degeneration is non-cell-autonomous, as neighboring glial cells, particularly astrocytes and microglia, can contribute to this process sustaining a vicious cycle of neuroinflammation [50]. Here, we have identified an evident up-regulation of S100A4 in the lumbar spinal cord of transgenic mutant SOD1 animals, starting from the pre-symptomatic phase of the disease up to the end-stage, which is particularly evident in astrocytes and microglia cells. These data are in accordance with the S100A4 up-regulation observed after brain injury in both humans and rodents [21] and, importantly, in astrocytes of pre-symptomatic SOD1-G37R mice [51]. Because the analysis of pre-symptomatic dysregulated genes is of extreme importance, as it can reveal pathways that might trigger or contribute directly to pathogenic mechanisms of the disease, S100A4 could represent a possible candidate in the early pathogenic mechanisms involved in ALS. In traumatic brain injury, S100A4 has neuroprotective functions [21], suggesting that also in our model of neurodegeneration, the protein could elicit a trophic role. Yet, many inflammatory molecules produced by glia in chronic neurodegenerative disease, and particularly in ALS, can have different effects on neuronal viability according to the disease phase (i.e., pre-symptomatic versus clear symptomatic) [52]. Thus, we cannot exclude that S100A4 can have protective functions in the early phases of the disease, switching into toxic roles when its expression is further increased at end-stage. On this matter, it would be very important to analyze if S100A4 is released by astrocytes/microglia during the disease and, therefore, can drive a stage-specific neuroinflammatory response. Furthermore, in analogy with its functions in inflammation and motility, S100A4 up-regulation in ALS-affected spinal cord could be potentially correlated to these features in the CNS, such as the massive migration of astrocytes and microglia towards the site of injury [53] and to the formation of the glial scar [20] and reactive gliosis, which characterize ALS-affected tissues [54]. Remarkably, at both pre-symptomatic and end-stage phases of the disease, we observed a strong increase of mTOR levels in SOD1-G93A animals that parallels the increased levels reported for the master regulator of inflammation NF-κB in ALS models [55,56]. In light of this, future experiments will be aimed at investigating whether the administration of niclosamide at different doses and different times could attenuate neuroinflammation and ameliorate symptoms in SOD1-G93A animal models.

In many neurodegenerative diseases, including ALS, several features of the CNS pathological modifications are also similarly present in patients’ peripheral cells, such as fibroblasts. Recent papers have indeed demonstrated that fibroblasts from both familial and sporadic ALS cases show numerous abnormalities, among which the metabolic alterations are indicative of oxidative stress and a pro-inflammatory state [57,58,59], sharing common pathogenic pathways with motor neurons [60,61,62]. In this work, we have demonstrated that S100A4 is also up-regulated in patient-derived fibroblasts bearing mutant *SOD1* in a single copy. Remarkably, in parallel with S100A4 increase, we also detected an increase in both mTOR and NF-κB proteins, indicating the concomitant alteration of these pathways, as in the case of microglial cells, supporting, therefore, the use of fibroblasts to study inflammatory functions. In addition, these data may propose S100A4 as a possible candidate biomarker of the disease, even though further investigations will be needed to evaluate its expression in fibroblasts from patients carrying other ALS-related mutations as well as in the sporadic cases. Although an association between S100A4 expression in fibroblasts and its expression in the CNS still needs to be clarified [37], the protein could represent a shared dysregulated factor by different cell types in ALS pathological mechanisms.

In conclusion, we identified a new pathway that appears to be aberrantly regulated in inflammatory environments as those occurring in ALS, and niclosamide as a possible drug to be applied in the attenuation of reactive phenotypes of microglia in uncontrolled pro-inflammatory conditions.

## Figures and Tables

**Table 1:** Clinical and demographic characteristics of patients.

Patient	Sex	Age of Onset	Site of Onset	Outcome	Disease Duration (Months)	Phenotype	Familial ALS	Disease Duration at Biopsy (Months)
SOD1 L84F	F	43	SLL	Tracheostomy and invasive ventilation	54	Pure LMN	Yes	13
SOD1 D90A	F	57	SLL	Alive	158	Classic	No	84
SOD1 G93D	M	56	SUL	Alive	100	Classic	Yes	29

SLL: spinal lower limb; SUL: spinal upper limb; Classic: both upper and lower motor neuron signs; Disease duration: refers to months from onset to the last follow-up in living patient or death or tracheostomy and invasive ventilation; Phenotype: refers to the clinical picture in the initial stage of disease, LMN: lower motor neuron signs without pyramidal involvement, ALS: amyotrophic lateral sclerosis.
